# Choosing important health outcomes for comparative effectiveness research: 4th annual update to a systematic review of core outcome sets for research

**DOI:** 10.1371/journal.pone.0209869

**Published:** 2018-12-28

**Authors:** Elizabeth Gargon, Sarah L. Gorst, Nicola L. Harman, Valerie Smith, Karen Matvienko-Sikar, Paula R. Williamson

**Affiliations:** 1 MRC North West Hub for Trials Methodology Research, Department of Biostatistics, University of Liverpool, Liverpool, United Kingdom; 2 School of Nursing and Midwifery, Trinity College Dublin, Dublin, Ireland; 3 School of Public Health, University College Cork, Cork, Ireland; University of Aberdeen Institute of Applied Health Sciences, UNITED KINGDOM

## Abstract

**Background:**

The Core Outcome Measures in Effectiveness Trials (COMET) database is a publically available, searchable repository of published and ongoing core outcome set (COS) studies. An annual systematic review update is carried out to maintain the currency of database content.

**Methods:**

The methods used in the fourth update of the systematic review followed the same approach used in the original review and previous updates. Studies were eligible for inclusion if they reported the development of a COS, regardless of any restrictions by age, health condition or setting. Searches were carried out in March 2018 to identify studies that had been published or indexed between January 2017 and the end of December 2017.

**Results:**

Forty-eight new studies, describing the development of 56 COS, were included. There has been an increase in the number of studies clearly specifying the scope of the COS in terms of the population (n = 43, 90%) and intervention (n = 48, 100%) characteristics. Public participation has continued to rise with over half (n = 27, 56%) of studies in the current review including input from members of the public. The rate of inclusion of all stakeholder groups has increased, in particular participation from non-clinical research experts has risen from 32% (mean average in previous reviews) to 62% (n = 29). Input from participants located in Australasia (n = 17; 41%), Asia (n = 18; 44%), South America (n = 13; 32%) and Africa (n = 7; 17%) have all increased since the previous reviews.

**Conclusion:**

This update included a pronounced increase in the number of new COS identified compared to the previous three updates. There was an improvement in the reporting of the scope, stakeholder participants and methods used. Furthermore, there has been an increase in participation from Australasia, Asia, South America and Africa. These advancements are reflective of the efforts made in recent years to raise awareness about the need for COS development and uptake, as well as developments in COS methodology.

## Introduction

Insufficient consideration of the outcomes measured in clinical trials, and other studies, is increasingly being addressed through the development and use of core outcome sets (COS). A COS is an agreed standardised set of outcomes that should be measured and reported, as a minimum, in all trials for a specific clinical area [1]. The Core Outcome Measures in Effectiveness Trials (COMET) Initiative aims to collate and stimulate the development and application of COS, by maintaining a public repository of studies relevant to the development of COS (The COMET database, http://www.comet-initiative.org/studies/search). A survey of users of the database was undertaken to understand the reasons why people were searching the COMET database [2]. The most frequent users were people thinking about developing a COS to see whether a COS already exists in their area of interest to avoid any duplication, therefore emphasising the importance of keeping the database current.

A systematic review was conducted to initially populate the COMET database [3], and it has been subsequently updated annually to include all published COS up to, and including, 2016 [2, 4, 5]. The previous update showed that COS developers are adopting a more structured approach towards COS development, and public participation in COS development has also increased [5]. The review, however, highlighted a gap in the involvement of stakeholders from a wider range of geographical settings, in particular low and middle income countries (LMIC). This is important to increase the applicability of COS to global health and tackling the global burden of disease [6].

The database is therefore an integral resource to not only the development of COS, but also to the uptake of COS in research and in the avoidance of unnecessary duplication and waste of scarce resources. Eligible studies are added to the database as they are found; however, an additional annual update to the systematic review means that the database is kept up to date.

The aims of the current study were consistent with the previous update [5], specifically to: (i) update the systematic review in order to identify any further studies where a COS has been developed; (ii) to describe the methodological approaches taken in these studies, and (iii) to highlight areas for future COS development and improvement.

## Methods

### Systematic review update

The methods used in this updated review followed the same approach used in the original review [3] and previous updates [2, 4, 5]. The methods were described in detail previously [3, 5]; a summary of methods is provided below.

### Study selection

The inclusion and exclusion criteria were described in detail in the original systematic review [3]. Studies were eligible for inclusion if they had applied methodology for determining which outcome domains or outcomes should be measured in clinical trials or other forms of health research. As described previously, studies were eligible for inclusion if they reported the development of a COS, regardless of any restrictions by age, health condition or setting. Studies describing the development of a Patient Reported Outcome (PRO) COS (a core set of patient-reported symptoms and health related quality of life domains) or Core Event Set (a core set of adverse events or complications) were eligible for inclusion in the review update. Studies describing the update of an existing COS were included as linked papers to the original COS.

### Identification of relevant studies

In March 2018, MEDLINE via Ovid and SCOPUS were searched without language restrictions. The search identified studies that had been published or indexed since the previous systematic review update, between January 2017 and the end of December 2017. The multifaceted search strategy, developed for the original review using a combination of text words and index terms, was used for the current update [3] (S1 Table). Ovid included a new search option entitled ‘MEDALL’ that incorporates all previous search options and provides the most extensive coverage, including the ‘in-process’ citations as well as E-pub ahead of print citations. This search option was selected for this update of the systematic review in order to identify eligible studies at the earliest possible opportunity.

Hand searching was completed, including any studies that had been submitted directly to the COMET database, references cited in eligible studies, as well as those in ineligible studies that referred to or used a COS.

### Selecting studies for inclusion in the review

As described previously [3, 5], records from each database were combined and duplicates removed. Titles and abstracts were read to assess eligibility of studies for inclusion in the review (stage 1). Full texts of potentially relevant articles were obtained to assess for inclusion (stage 2). Two of five reviewers (EG, SG, NH, VS and KMS) independently checked the title and abstract of each citation. Citations were retained for further checking if agreement could not be reached. Two of the same five reviewers assessed each full paper for inclusion in the review. In cases of disagreement a third reviewer was consulted. The reasons for exclusion at this stage were documented for articles judged to be ineligible.

### Checking for agreement between reviewers

One reviewer was new to this systematic review and had not been involved in previous updates (KMS). During each stage of the review process, agreement between KMS and the lead reviewer (EG) was assessed prior to independently assessing records. Agreement between all other reviewers had been checked in previous updates.

### Checking for correct exclusion

#### At abstract stage

A 1% sample of the records excluded on the basis of the title and abstract was checked by a sixth reviewer (Jamie Kirkham) and assessed for correct exclusion. If any studies were identified as being incorrectly excluded, further checking was performed within the other excluded records.

#### At full text stage

Of the records that had been excluded after reading the full text, 5% were assessed for correct exclusion. If any studies were identified as being incorrectly excluded at this stage, further checking was performed.

### Data extraction

Described in full previously [3], data were extracted in relation to the study aim(s), health area, target population, interventions covered, methods of COS development and stakeholder groups involved. Data relating to the geographical locations of participants included in the development of COS were also extracted.

### Data analysis and presentation of results

We report the review in accordance with PRISMA guidelines [7] (S1 File). We describe the studies narratively, and present the findings in text and tables. As in previous updates, we did not anticipate conducting any statistical analyses to combine the findings.

## Results

### Description of studies

**Fig 1:**
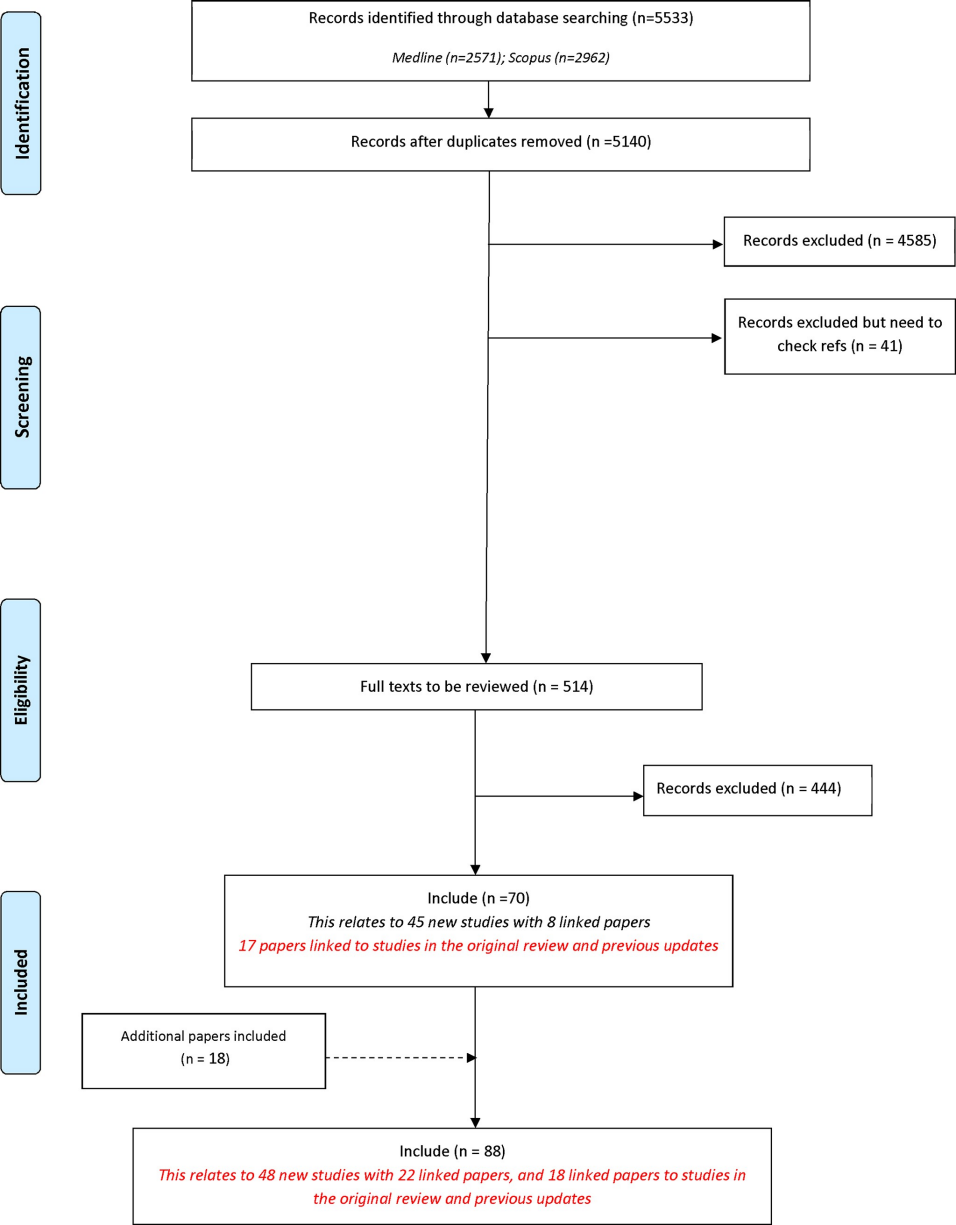
Identification of studies.

Following the removal of duplicates, 5140 records were identified in the database search. A total of 4626 records were excluded during the title and abstract stage, and a further 444 were excluded following the assessment of full text (Fig 1). Table 1 provides a summary of the reasons for exclusion of records at full text stage. Seventy records related to 45 new studies that met the inclusion criteria. In addition to the review search, three additional records were identified through database search alerts as being eligible for inclusion in the review. These three studies were not identified during the review search, as although they were published in 2017, they had not been indexed in the databases at the time we ran our search. A further 15 reports were identified by hand searching references of included studies. In total, 88 reports relating to 48 new studies describing the development of 56 COS were included for the first time in this update (S2 Table).

**Table 1:** Reasons for exclusion at full text stage.

Exclusion Categories of Full Text Stage	Number of records
Studies relating to how, rather than which, outcomes should be measured	50
Studies reporting the design/ rationale of single trial	3
Studies reporting the use of a COS	1
Systematic reviews of clinical trials	39
Review/overview/discussion only, no outcome recommendations	113
Core outcomes/ outcome recommendations not made	72
Quality indicators	5
One outcome/ domain only	8
Instrument development	8
Recommendations by single author only	2
ICF Core set	1
Preclinical/ Early phase only (0, I, II)	8
Irrelevant	43
Assessed in previous review	3
ICF Core set development	2
HRQL	3
Recommendations for clinical management in practice not research	61
Studies that elicit stakeholder group opinion regarding which outcome domains or outcomes are important	8
Ongoing studies	14

### Included studies

#### Year of publication

**Fig 2:**
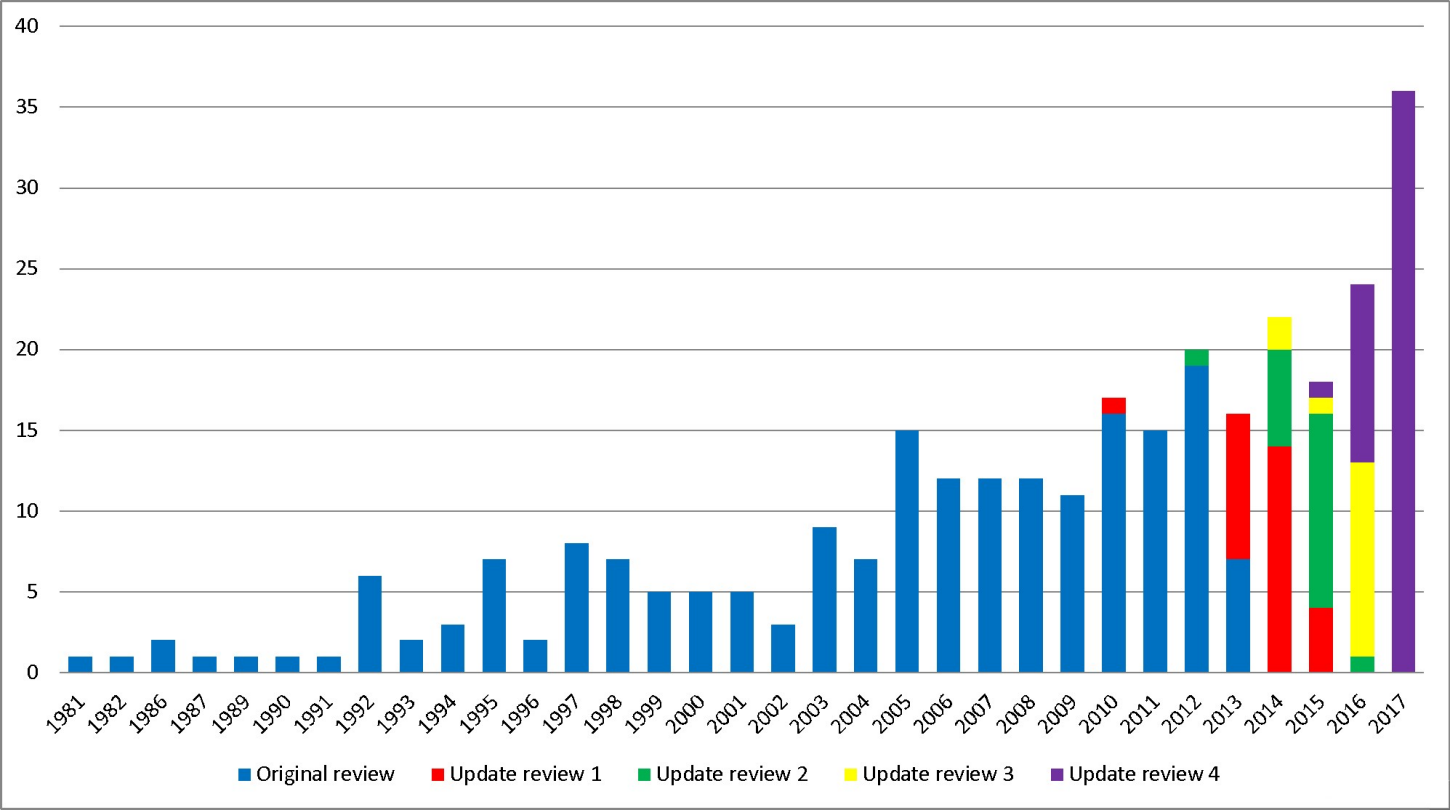
Year of first publication of each COS study (n = 307).

The year of first publication of COS has been updated to include the 48 new studies included in this update (Fig 2). Of the 48 studies identified in this update, 36 were published in 2017, 11 were published in 2016 and one study was published in 2015.

#### Scope of core outcome sets

**Fig 3:**
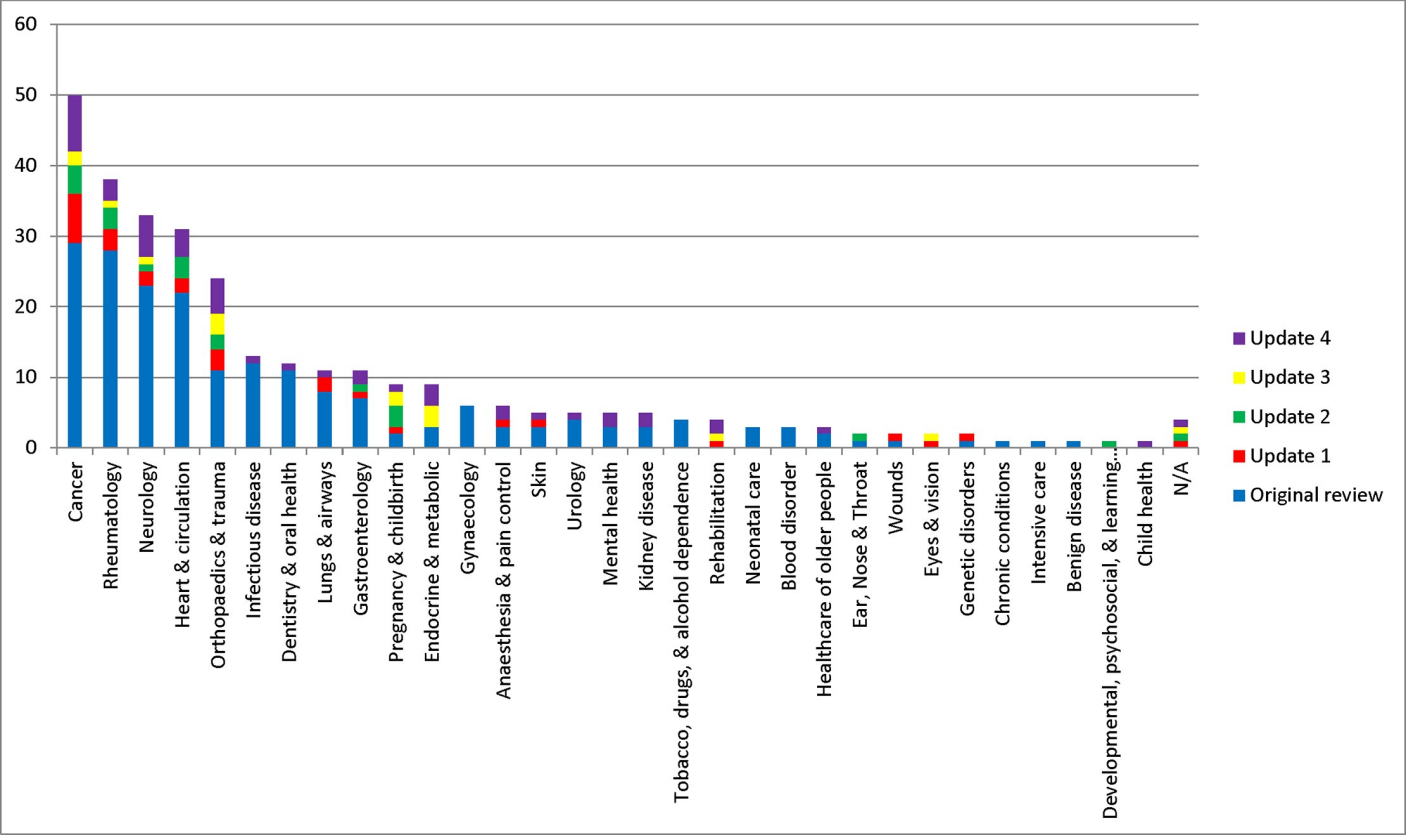
Number of COS developed in each disease category (n = 307).

The scope of published COS studies is summarised in Table 2. This includes study aims, setting for intended use, population characteristics and intervention characteristics. Table 2 highlights that there has been an increase in the number of COS studies specifying the population (n = 43, 90%) and intervention (n = 48, 100%) characteristics of the newly developed COS. Fig 3 displays the number of COS that have been developed in each disease category. The majority of COS have been developed within the areas of cancer, rheumatology, heart & circulation, neurology and orthopaedics & trauma. Disease categories and disease names are provided in S2 Table.

**Table 2:** The scope of included studies (n = 307).

	Original review n (%)	Update review 1 n (%)	Update review 2 n (%)	Update review 3 n (%)	Update review 4 n (%)	Combined* N (%)
**Study aims**						
Specifically considered outcome selection and measurement	98 (50)	21 (75)	13 (65)	10 (60)	33 (69)	175 (57)
Considered outcomes while addressing wider clinical trial design issues	98 (50)	7 (25)	7 (35)	5 (40)	15 (31)	132 (43)
**Intended use of recommendations**						
Clinical research	174 (89)	24 (86)	19 (95)	9 (60)	44 (92)	270 (88)
Clinical research and practice	22 (11)	4 (14)	1 (5)	6 (40)	4 (8)	37 (12)
**Population characteristics**						
Adults	12 (6)	12 (43)	5 (25)	10 (67)	21 (44)	63 (21)
Children	22 (11)	2 (7)	6 (30)	0 (0)	5 (10)	35 (11)
Adults and children	12 (6)	2 (7)	0 (0)	3 (20)	10 (21)	27 (9)
Older adults	2 (1)	1 (4)	0 (0)	0 (0)	3 (6)	6 (2)
Adolescents and adults	0 (0)	0 (0)	0 (0)	1 (7)	4 (8)	5 (2)
Not specified	148 (76)	11 (39)	9 (45)	2 (13)	5 (10)	171 (56)
**Intervention characteristics**						
All intervention types	7 (4)	8 (29)	12 (60)	8 (53)	29 (60)	68 (22)
Drug treatments	39 (20)	4 (14)	0 (0)	0 (0)	4 (8)	46 (15)
Surgery	13 (7)	4 (14)	4 (20)	4 (27)	7 (15)	32 (10)
Vaccine	2 (1)	0 (0)	0 (0)	0 (0)	0	2 (1)
Rehabilitation	1 (1)	1 (4)	0 (0)	1 (7)	2 (4)	5 (2)
Exercise	1 (1)	1 (4)	1 (5)	0 (0)	1 (2)	4 (1)
*Exercise (physical activity)*	*1*	*0*	*1*	*0*	*1*	
*Exercise (yoga)*	*0*	*1*	*0*	*0*	*0*	
Procedure	4 (2)	0 (0)	2 (10)	0 (0)	2 (4)	8 (3)
Device	3 (2)	0 (0)	0 (0)	1 (7)	0	4 (1)
Other	11 (6)	5 (18)	0 (0)	1 (7)	3 (6)	20 (7)
Not specified	115 (59)	5 (18)	1 (5)	0 (0)	0	118 (38)

*Additional information provided by updated papers linked to previously published COS are reflected in the combined column.

#### Methods used to select outcomes

The methods used to develop the 48 new COS identified in the current review are presented in Table 3 alongside the methods used in the four previous systematic reviews. Table 3 highlights that the use of mixed methods to develop a COS has remained high (n = 35; 73%). The use of the Delphi technique has also remained high with 48% (n = 23) of studies using the technique in combination with other methods.

**Table 3:** The methods used to develop COS (n = 307).

Main methods	Original review n (%)	Update review 1 n (%)	Update review 2 n (%)	Update review 3 n (%)	Update review 4 n (%)	Combined* N (%)
**Semi-structured group discussion only**	55 (28)	2 (7)	2 (10)	0 (0)	3 (6)	62 (20)
**Unstructured group discussion only**	18 (9)	0 (0)	0 (0)	0 (0)	0 (0)	18 (6)
**Consensus development conference only**	12 (6)	0 (0)	1 (5)	0 (0)	1 (2)	14 (5)
**Literature/systematic review only**	11 (6)	5 (18)	2 (10)	1 (7)	6 (13)	25 (8)
**Delphi only**	6 (3)	2 (7)	2 (10)	0 (0)	0 (0)	10 (3)
**Survey only**	3 (2)	0 (0)	0 (0)	0 (0)	1 (2)	4 (1)
**NGT only**	1 (1)	0 (0)	0 (0)	0 (0)	0 (0)	1 (<1)
**Interview only**	0 (0)	0 (0)	0 (0)	0 (0)	1 (2)	1 (<1)
**Mixed methods *(see descriptions below*)**	74 (38)	17 (61)	13 (65)	12 (80)	35 (73)	151 (49)
*Delphi + another method(s)*	*22 (11)*	*6 (21)*	*9 (45)*	*9 (60)*	*23 (48)*	*71 (23)*
*Semi-structured group discussion + another method(s)*	*30 (15)*	*7 (25)*	*4 (20)*	*2 (13)*	*9 (19)*	*51 (17)*
*Consensus development conference + another method(s)*	*7 (4)*	*0 (0)*	*0 (0)*	*0 (0)*	*1 (2)*	*8 (3)*
*Literature/systematic review + another method(s)*	*10 (5)*	*4 (14)*	*0 (0)*	*1 (7)*	*2 (4)*	*16 (5)*
*NGT + another method(s)*	*4 (2)*	*0 (0)*	*0 (0)*	*0 (0)*	*0 (0)*	*4 (1)*
*Focus group + another method(s)*	*1 (1)*	*0 (0)*	*0 (0)*	*0 (0)*	*0 (0)*	*1 (<1)*
No methods described	16 (8)	2 (7)	0 (0)	2 (13)	1 (2)	21 (7)

*Additional information provided by updated papers linked to previously published COS are reflected in the combined column.

#### People involved in selecting outcomes

The list of stakeholders included in COS development (Table 4) has been updated to include the COS identified in this update. Of the 307 published COS studies, 273 (89%) provided information about the stakeholders who participated in the COS development. Clinical experts were included in 268 (98%) studies; this is in direct contrast to public representatives who were included in 89 (33%) studies. Public participation has continued to rise in recent years, with 56% (n = 27) of studies in the current review including input from members of the public. The rate of inclusion of all stakeholder groups has increased in comparison to the original review and previous review updates, with participation from non-clinical research experts, in particular, rising from 32% (n = 73) as the mean average in previous reviews to 62% (n = 29) in the current review.

**Table 4:** Participant groups involved in selecting outcomes for inclusion in COS (n = 307).

Participants category	Sub-category (not mutually exclusive)	Frequency of participants					
		Original review n (%)	Update review 1 n (%)	Update review 2 n (%)	Update review 3 n (%)	Update review 4 n (%)	Combined^ N (%)
**Clinical experts**		**171 (99)**	**20 (95)**	**17 (100)**	**14 (93)**	**46 (96)**	**268 (98)**
	Clinical experts	86	14	16	14	38 (79)	169 (62)
	Clinical research expertise	66	9	9	2	26 (54)	114 (42)
	Clinical trialists/ Members of a clinical trial network	9	2			1 (2)	12 (4)
	Others with assumptions*	54					54 (20)
**Public representatives**		**30 (17)**	**13 (62)**	**11 (65)**	**8 (53)**	**27 (56)**	**89 (33)**
	Patients	19	11	7	8	18 (38)	63 (23)
	Carers	7	1	3	3	9 (19)	23 (8)
	Patient support group representatives	9	1	4		9 (19)	24 (9)
	Service users	2			1	2 (4)	5 (2)
**Non-clinical research experts**		**53 (31)**	**9 (43)**	**9 (53)**	**2 (13)**	**29 (60)**	**103 (38)**
	Researchers	26	4	4	2	26 (54)	64 (23)
	Statisticians	19	4	3		1 (2)	27 (10)
	Epidemiologists	11	2	1		4 (8)	19 (7)
	Academic research representatives	4					4 (2)
	Methodologists	6	3	2		4 (8)	16 (6)
	Economists	3		1		2 (4)	6 (2)
**Authorities**		**39 (23)**	**5 (24)**	**3 (18)**	**0 (0)**	**12 (25)**	**59 (22)**
	Regulatory agency representatives	30	4	3		6 (13)	43 (16)
	Governmental agencies	12	1			5 (10)	18 (7)
	Policy makers	4	1			3 (6)	8 (3)
	Charities	1				1 (2)	2 (1)
	Service commissioners					3 (6)	3 (1)
**Industry representatives**		**31 (18)**	**4 (19)**	**3 (18)**	**0 (0)**	**9 (19)**	**47 (17)**
	Pharmaceutical industry representatives	28	3	3		8 (17)	42 (15)
	Device manufacturers	2	1			1 (2)	4 (2)
	Biotechnology company representatives	1					1 (<1)
**Others**		**72 (42)**	**2 (10)**	**1 (6)**	**1 (7)**	**8 (17)**	**84 (31)**
	Service providers					4 (8)	4 (2)
	Ethicists	1					1 (<1)
	Journal editors	2		1		2 (4)	5 (2)
	Funding bodies		1				1 (<1)
	Yoga therapists/ instructors		1				1 (<1)
	Members of health care transition research consortium				1		1 (<1)
	Educationalist					1 (2)	1 (<1)
	Nutritionist					1 (2)	1 (<1)
	National professional and academic bodies/ committees					1 (2)	1 (<1)
	Others** (besides known participants)	15					15 (6)
	Others with assumptions*	54					54 (20)
**No details given**		**24 (12)**	**7 (25)**	**3 (15)**	**0 (0)**	**0 (0)**	**34 (11)**

* 54 studies with clinical input but unclear about involvement of other stakeholders

** Workshop/meeting participants (*5), subcommittee/committee (*2), guidelines panel, military personnel, moderator and audience, representatives from EORTC, members with expertise in information technologies, informatics, clinical registries, data-standards development, expertise in vaccine safety, malaria control and representatives from funding agencies/registration authorities, and donor organisation, members of the Rheumatology Section of the American Academy of Pediatrics, the Pediatric Section of the ACR, and the Arthritis Foundation, the diagnostic radiology and basic science communities, and from individuals conversant with functional and quality of life (QOL) assessments, comparative effectiveness research, and cost/ benefit analysis

^Additional information provided by updated papers linked to previously published COS are reflected in the combined column

As described previously [3, 5], public representatives include patients, carers, health and social care service users and people from organisations who represent these groups. The degree of public participation within the development of the COS studies included in this updated review is described in Table 5. All of the 27 studies that reported including public participants provided some detail about their participation. Among Delphi studies that reported participation of both clinical experts and the public, levels of participation for public participants ranged from 6% [8] to 69% [9].

**Table 5:** Nature of patient participation where detail is reported (n = 27).

	Methods used	Total number of participants	Number of public participants	% Public participants when multiple stakeholder groups included
**Agiostratidou**	Survey	Not reported	8	Unknown
**Allin**	Delphi	Round 1: 108	Round 1: 61	57%
		Round 2: 96	Round 2: 51	53%
		Round 3: 89	Round 3: 46	52%
	Consensus meeting	17	6	35%
	Measurement meeting	14	4	29%
**Ammendolia**	Interviews*	28	28	
**Avery**	Interviews	38	31	82%
	Delphi	Round 1: 188	Round 1: 116	62%
		Round 2: 161	Round 2: 94	58%
	Consensus meeting*	20	20	
**Benstoem**	Delphi	Round 1: 86	Round 1: NR	16%
		Round 2: 46	Round 2: NR	16%
		Round 3: 39	Round 3: NR	16%
**Byrne**	Delphi	Round 1: 132	Round 1: 34	26%
		Round 2: 81	Round 2: 17	21%
	Consensus meeting	12	3	25%
**de Graaf**	Interviews*	26	26	
	Delphi^	Round 1: 126	Round 1: 0	
		Round 2: 97	Round 2: 0	
**Egan**	Delphi	Round 1: 151	Round 1: 20	13%
		Round 2: 120	Round 2: NR	Unknown
		Round 3: 101	Round 3: NR	Unknown
	Consensus meeting	14	2	14%
**Grieve**	Workshops (x4)	W1: 27	W1: 5	19%
		W2: 15	W2: 1	7%
		W3: 20	W3: 5	25%
		W4: 18	W4: 3	17%
**Klokkerud**	Delphi consensus meeting	46	6	13%
**Layton**	Delphi	Round 1: 500	Round 1: 307	61%
		Round 2: 164	Round 2: 52	32%
		Round 3: 116	Round 3: 34	29%
**MacLennan**	Interviews*	15	15	
	Delphi	Round 1: 174	Round 1: 118	68%
		Round 2: 158	Round 2: 109	69%
		Round 3: 152	Round 3: 105	69%
	Meeting	21	8	38%
**Marrie**	Survey	296	20	7%
	Workshop	Not reported	Not reported	unknown
**McNamara**	Delphi	17	1	6%
**Millar**	Focus groups	72	14	19%
	Interviews^	13	0	
	Delphi	Round 1: 19	Round 1: 2	11%
		Round 2: 18	Round 2: NR	
**Nabbout**	Interviews	11	7	64%
	Delphi^	Round 1: 8	Round 1: 0	
		Round 2: 7	Round 2: 0	
**Nikiphorou**	Workshops and teleconference	26	2	8%
**Obbarius**	Delphi	24	2	8%
	Interviews	not reported	not reported	
**Ong**	Focus groups*	8	8	
	Survey*	1225	1225	
	Delphi	not reported	not reported	
**Page**	Delphi	Round 1: 91	Round 1: 41	45%
		Round 2: 96	Round 2: 41	43%
**Sreih**	Delphi^	99	0	
	Interviews/focus groups*	31	31	
**Steutel**	Delphi	Round 1: 188	Round 1: 55	
		Round 2: 97	Round 2: 43	
	Meeting^	not reported	0	
**Tong**	NGT/focus groups*	57	57	
	Delphi	Round 1: 1018	Round 1: 461	45%
		Round 2: 844	Round 2: 387	46%
		Round 3: 779	Round 3: 360	46%
	Workshops	124	27	22%
**Turnbull**	Survey	279	158	57%
	Delphi	77	19	25%
	Interviews*	48	48	
	Survey^	178	0	
	Meeting^	129	0	
**van der Poel**	Delphi^	Round 1: 30	Round 1: 30	
		Round 2: 30	Round 2: 30	
	Consensus meeting	16	1	6%
**Wallace**	NGT*	68	68	
	Delphi^	Round 1: 318	Round 1: 0	
		Round 2: 153	Round 2: 0	
		Round 3: 137	Round 3: 0	
	Delphi^^	Round 1: 72	Round 1: 0	
		Round 2: 63	Round 2: 0	
		Round 3: 61	Round 3: 0	
**Webster**	Focus groups*	18	18	
	Consensus conference^	27	0	
	Consensus conference^	27	0	

*patient only.

^clinician only.

^^researcher only.

Table 6 lists the geographical locations, by continent, of the participants involved in developing the COS in this update and in the combined reviews, as reported by the included studies. In the current review, participant locations were provided in 41 of the 48 studies, however for the remaining seven studies, location information was only provided for the authors. The majority of COS were developed with the inclusion of participants located in Europe (n = 38; 93%) and North America (n = 28; 68%). However, input from participants located in Australasia (n = 17; 41%), Asia (n = 18; 44%), South America (n = 13; 32%) and Africa (n = 7; 17%) has increased since the previous reviews. The number of countries that have been involved in the development of a COS ranges from 1 to 76 (a median of 6).

**Table 6:** Geographical locations of participants included in the development of each COS (n = 256).

Locations	Original review n (%)	Update review 1 n (%)	Update review 2 n (%)	Update review 3 n (%)	Update review 4 n (%)	Combined* N (%)
North America	134 (82)	17 (68)	9 (64)	6 (55)	28 (68)	196 (77)
Europe	125 (76)	19 (76)	13 (93)	10 (91)	38 (93)	206 (81)
Australasia	42 (26)	4 (16)	5 (36)	3 (27)	17 (41)	74 (29)
Asia	34 (21)	3 (12)	6 (43)	1 (9)	18 (44)	63 (25)
South America	16 (10)	3 (12)	2 (14)	1 (9)	13 (32)	37 (15)
Africa	10 (6)	1 (4)	2 (14)	1 (9)	7 (17)	22 (9)
Total	164 (84)	25 (89)	14 (70)	11 (73)	41 (85)	256 (83)
No details provided	32 (16)	3 (11)	6 (30)	4 (27)	7 (15)	51 (17)
Median and range of number of countries	6, 1–76	2, 1–33	6, 1–28	2, 1–18	6, 1–37	6, 1–76

*Additional information provided by updated papers linked to previously published COS are reflected in the combined column.

## Discussion

This systematic review update has identified 48 new COS articles that describe 56 new COS. This is a marked increase in the number of new COS identified in the previous three updates, which included 28, 20 and 15 new COS publications (describing the development of 29, 20 and 15 COS) respectively [2, 4, 5]. Similarly, there has been a continued increase in COMET website and database visits, new visitors, number of database searches and visits from around the world in 2017 (Source of data usage: Google Analytics), with 24,884 visits; 15,546 new visitors; 5142 database searches, and visitors from 139 countries. The sustained growth in use suggests that the COMET website and database are continuing to raise awareness about the existence of, and need for, COS; which could explain this increase in the number of COS published in 2017 and subsequently included in this review. Their inclusion in the database brings the overall total of COS publications to 307, relating to 366 COS.

This review has identified an improvement in the reporting of COS scope (population and intervention characteristics), stakeholder participants and methods used. Core Outcome Set-STAndards for Reporting (COS-STAR) were published in 2016 [10]. This is the first update since the publication of the COS-STAR guidelines, and it is encouraging to see that ten of the 48 studies (21%) included in this update referenced these reporting guidelines. This provides a baseline with which to compare future updates, where we hope to see a continued increase in the number of studies reporting their work using these guidelines.

The previous update highlighted an increase in the use of mixed methods, including the Delphi method, for COS development [5]. A recent survey of ongoing COS developers indicated that mixed methods, including Delphi, were commonly used in ongoing COS development, and there is an increased inclusion of public participants in ongoing studies [11]. As such, the percentage of new studies including public participation is expected to rise in future updates of this systematic review as ongoing studies are published. Participation of non-clinical research experts has increased, and as one of the criteria included in the recently published minimum standards for COS development [12], we would expect that this should continue to increase in new COS studies included in any further updates to this review. The Core Outcome Set-STAndards for Development (COS-STAD) recommendations will help users to assess whether a particular COS has been developed using a reasonable approach.

The involvement of stakeholders from a range of countries, but in particular lower and middle income countries, has previously been identified as a gap in COS development. [5] This update has shown an increase in participation from Australasia, Asia, South America and Africa. A recent survey to identify the top priorities for trials methodology research in LMICs found that choosing appropriate outcomes to measure was one of the two most important topics [6]. The involvement of LMIC countries in COS development and uptake is therefore a key area of importance and efforts should be made to improve this.

In conclusion, we have completed the fourth update to a systematic review of COS, identifying COS published and indexed in 2017. COS methodology, and consensus development methodology more generally, have developed over recent years. This is reflected in the improvements seen throughout these updates, not only in the reporting of important COS details (such as scope), but in the methods chosen for development and the stakeholders who participate in the process. With the recent development and publication of guidelines and minimum standards for COS development [1, 12], it is hoped that improvements in these areas will continue.

## Supporting information

S1 File PRISMA Checklist. PRISMA checklist for content of a systematic review.
(DOC)

S1 Table Search strategy. (DOCX)

S2 Table Table of reports included in updated review (n = 88). (DOCX)
