# Supplementary material for: Choosing important health outcomes for comparative effectiveness research: 4th annual update to a systematic review of core outcome sets for research
Source: PLoS One. 2018 Dec 28;13(12):e0209869. doi: 10.1371/journal.pone.0209869 (PMC6310275; doi:10.1371/journal.pone.0209869)
Supplement: S1 Table — (DOCX) [file pone.0209869.s002.docx]

**S1 Table. Search strategy**

| **Search terms for MEDLINE** | | | |
| --- | --- | --- | --- |
| 1 | | Health Services/ut [Utilization] | |
| 2 | | registries/ | |
| 3 | | systematic review.mp. | |
| 4 | | structured review.ti. | |
| 5 | | evidence based medicine.ab. | |
| 6 | | exp Clinical Trials as Topic/ | |
| 7 | | clinical trial$.ab. | |
| 8 | | randomised controlled trial$.ti,ab. | |
| 9 | | randomised trial$.ti,ab. | |
| 10 | | 1 or 2 or 3 or 4 or 5 or 6 or 7 or 8 or 9 | |
| 11 | | workgroup$.mp. | |
| 12 | | standard$ outcome$.mp. | |
| 13 | | Practice Guideline/ | |
| 14 | | clinical database.mp. | |
| 15 | | patient important outcome$.mp. | |
| 16 | | (standard$ adj3 reporting).mp. | |
| 17 | | congresses.pt. | |
| 18 | | Delphi Technique/ | |
| 19 | | (recommend$ adj3 outcome$).mp. | |
| 20 | | consensus development conference.pt. | |
| 21 | | outcome$ reporting.mp. | |
| 22 | | priorit$ symptom$.mp. | |
| 23 | | (task force adj3 outcome$).mp. | |
| 24 | | appropriate outcome$.mp. | |
| 25 | research design/ | |  |
| 26 | | endpoint determination/ | |
| 27 | | consensus development conference/ | |
| 28 | | patient participation/ | |
| 29 | | consensus.mp. | |
| 30 | | workshop.mp. | |
| 31 | | Consensus Development Conferences, NIH as Topic/ | |
| 32 | | focus groups/ | |
| 33 | | 11 or 12 or 13 or 14 or 15 or 16 or 17 or 18 or 19 or 20 or 21 or 22 or 23 or 24 or 25 or 26 or 27 or 28 or 29 or 30 or 31 or 32 | |
| 34 | | outcome$.mp. | |
| 35 | | end point$.mp. | |
| 36 | | (core adj3 set).mp. | |
| 37 | | treatment emergent problem$.mp. | |
| 38 | | exp outcome Assessment Health Care/ | |
| 39 | | Treatment Outcome/ | |
| 40 | | Quality of Life/ | |
| 41 | | 34 or 35 or 36 or 37 or 38 or 39 or 40 | |
| 42 | | clinical-study design.mp. | |
| 43 | | patient$ perspective$.ti. | |
| 44 | | outcome$.mp. and delphi.ti. | |
| 45 | | (outcome$ and delphi).ab. | |
| 46 | | (perspective$ adj3 outcome$).ti. | |
| 47 | | core outcome$.ti,ab. | |
| 48 | | core set$.ti,ab. | |
| 49 | | clinical trial design$.ti. | |
| 50 | | design$ clinical trial$.ti. | |
| 51 | | (consensus and outcome$).ti. | |
| 52 | | 42 or 43 or 44 or 45 or 46 or 47 or 48 or 49 or 50 or 51 | |
| 53 | | 10 and 33 and 41 | |
| 54 | | 52 or 53 | |
|  | | limit to ed=20170101-20171231 | |
| **Search terms for SCOPUS** | | | |
| ((((INDEXTERMS(registries)) OR (INDEXTERMS(clinical trials as topic)) OR (ABS("evidence based medicine")) OR (ABS("clinical trial*")) OR (INDEXTERMS("Health Services Utilization")) OR (TITLE-ABS-KEY("SYSTEMATIC REVIEW")) OR (TITLE("structured review"))) OR (TITLE OR ABS("randomised controlled trial*")) OR (TITLE OR ABS (randomised trial*))) AND (((TITLE-ABS-KEY(workgroup*)) OR (TITLE-ABS-KEY(standard* outcome*)) OR (INDEXTERMS(practice guideline)) OR (TITLE-ABS-KEY("clinical database")) OR (TITLE-ABS-KEY("patient important outcome*")) OR (TITLE-ABS-KEY("standard* outcome*")) OR (INDEXTERMS(delphi technique))) OR ((TITLE-ABS-KEY(recommend* W/3 outcome*)) OR (TITLE-ABS-KEY(standard* W/3 reporting*)) OR (TITLE-ABS-KEY(task force W/3 outcome*)) OR (TITLE-ABS-KEY("appropriate outcome*")) OR (TITLE-ABS-KEY("outcome* reporting")) OR (TITLE-ABS-KEY("priorit* symptom*")) OR (INDEXTERMS(focus group)) (INDEXTERMS(research design))) OR ((INDEXTERMS(endpoint determination)) OR (INDEXTERMS(consensus development conference)) OR (INDEXTERMS(patient participation)) OR (TITLE-ABS-KEY(consensus)) OR (TITLE-ABS-KEY(workshop)))) AND 74) OR (((TITLE("design* clinical trials")) OR (TITLE(consensus AND outcome*)) OR (TITLE-ABS-KEY("clinical-study design")) OR (TITLE("patient* perspective*")) OR (ABS(outcome* AND delphi)) OR (TITLE(outcome* AND delphi)) OR (TITLE(perspective* W/3 outcome*)) OR (ABS("core outcome*") OR TITLE("core outcome*"))) OR ((ABS("core set*") OR TITLE("core set*")) OR (TITLE("clinical trial design*")))) **AND  ORIG-LOAD-DATE  >  *20170101*  AND  ORIG-LOAD-DATE  <  *20171231*** | | | |
