# Supplementary material for: Choosing important health outcomes for comparative effectiveness research: 4th annual update to a systematic review of core outcome sets for research
Source: PLoS One. 2018 Dec 28;13(12):e0209869. doi: 10.1371/journal.pone.0209869 (PMC6310275; doi:10.1371/journal.pone.0209869)
Supplement: S2 Table — (DOCX) [file pone.0209869.s003.docx]

**S2 Table. Table of reports included in updated review (n=88)**

| **Study** | **Disease category** | **Disease name** |
| --- | --- | --- |
| Agiostratidou 2017 [1]** | Endocrine & metabolic | Type 1 Diabetes |
| Allin 2017 [2]**  Allin 2016 [3] | Gastroenterology | Hirschsprung's disease |
| Ammendolia 2017 [4]** | Orthopaedics & trauma | Neurogenic claudication |
| Askenazi 2016 [5]* | Kidney disease | Neonatal acute kidney injury |
| Avery 2018 [6]**  Blencowe 2012 [7] | Cancer | Esophageal cancer |
| Barbarot 2016 [8]^  Chalmers 2016 [9]  Gerbens 2016 [10]  Gerbens 2016 [11]  Heinl 2017 [12]  Schmitt 2013 [13]  Spuls 2017 [14] | Skin | Atopic eczema/dermatitis |
| Benstoem 2017 [15]**  Benstoem 2015 [16] | Heart & circulation | Cardiovascular disease |
| Byrne 2017 [17]** | Endocrine & metabolic | Type 1 Diabetes |
| Chiarotto 2016 [18]^ | Orthopaedics & trauma | Low back pain |
| Christiansen 2016 [19]* | Endocrine & metabolic | N/A |
| de Graaf 2017 [20]** | Orthopaedics & trauma; Rheumatology | Acute osteomyelitis or septic arthritis - paediatric bone and joint infections |
| Dohner 2017 [21]*  Dohner 2010 [22] | Cancer | Acute myeloid leukemia |
| Egan 2017 [23]** | Pregnancy and childbirth | Pregestational diabetes |
| Flint 2017 [24]** | Neurology | Epilepsy |
| Gan 2017 [25]^ | Skin | Vitiligo |
| Garcia-Cardenas 2016 [26]** | Lungs & airways | Asthma |
| Glynne-Jones 2017 [27]^ | Cancer | Anal cancer |
| Grieve 2017 [28]** | Anaesthesia & pain control | Complex regional pain syndrome |
| Harris 2017 [29]** | Infectious disease | Bloodstream infections (BSI) |
| Hernandez Yenty 2016 [30]* | Other | Benign inverted nipple |
| Højgaard 2017 [31]^  Ogdie 2017 [32]  Kalyoncu 2016 [33]  Orbai 2017 [34] | Rheumatology | Psoriatic arthritis |
| Kamat 2016 [35]* | Cancer | Non–muscle-invasive bladder cancer |
| Karam 2017 [36]* | Cancer | Ovarian cancer |
| Kenny 2018 [37]**  Sharif 2015 [38] | Dentistry & oral health | Traumatic dental injuries |
| Kilari 2016 [39]* | Cancer; Health care of older adults | Cancer |
| Klokkerud 2018 [40]** | Rehabilitation, Rheumatology | Musculoskeletal diseases |
| Kwakkel [41]** | Neurology; Rehabilitation | Stroke |
| Layton 2017 [42]** | Skin | Acne |
| Mackie 2017 [43]^ | Rheumatology | Polymyalgia rheumatica |
| MacLennan 2017 [44]** | Cancer | Prostate cancer |
| Marrie 2016 [45]* | Neurology | Multiple sclerosis |
| McNamara 2015 [46]** | Heart & circulation | Coronary artery disease |
| Millar 2017 [47]** | Healthcare of older people | N/A |
| Murray 2017 [48]* | Orthopaedics & trauma | Musculoskeletal injuries |
| Nabbout 2018 [49]** | Neurology | Dravet Syndrome |
| Nikiphorou 2017 [50]* | Rheumatology | Rheumatoid arthritis |
| Obbarius 2017 [51]** | Mental health | Depression and anxiety |
| Ong 2017 [52]** | Cancer | Breast cancer |
| Page 2016 [53]**  Buchbinder 2017 [54]  Page 2018 [55] | Orthopaedics & trauma | Shoulder disorders |
| Rief 2017 [56]** | Mental health | Somatic symptom disorders |
| Robson 2017 [57]^ | Rheumatology | Antineutrophil cytoplasmic antibody-associated vasculitis |
| Ruiz 2017 [58]** | Heart & circulation | N/A |
| Sanderson 2016 [59]^ | Rheumatology | Rheumatoid arthritis |
| Sharrock 2016 [60]* | Orthopaedics & trauma | N/A |
| Sreih 2016 [61]** | Rheumatology | Large-vessel vasculitis |
| Steutel 2017 [62]**  Steutel 2014 [63] | Child health | Infant colic |
| Stoffel 2017 [64]* | Urology | Nonneurogenic chronic urinary retention |
| Stoner 2016 [65]* | Heart & circulation | Chronic lower extremity peripheral artery disease |
| Tong 2017 [66]**  Sautenet 2017 [67]  Howell 2012 [68]  Howell 2016 [69] | Kidney disease | Kidney transplant |
| Turnbull 2017 [70]**  Needham 2017 [71]  Dinglas 2018 [72]  Hashem 2016 [73]  Turnbull 2016 [74]  Hodgson 2017 [75]  Eakin 2017 [76] | Rehabilitation | Respiratory failure |
| van der Poel 2017 [77]*  Acar 2015 [78] | Cancer | Prostate cancer |
| Walker 2017 [79]** | Rheumatology | Systemic sclerosis |
| Wallace 2017a [80]**  Wallace 2017b [81]  Wallace 2017c [82]  Wallace 2016 [83] | Neurology | Aphasia |
| Warners 2017 [84]** | Gastroenterology | Eosinophilic esophagitis |
| Webster 2017a [85]**  Webster 2017b [86] | Neurology | Dementia |
| Williams 2017 [87]** | Anaesthesia & pain control | N/A |
| Wolters 2016 [88]^ | Genetic disorders | Neurofibromatosis |

*^ Linked to COS included in previous review*

** Considered outcomes while addressing wider clinical trial design issues*

*** Specifically considered outcome selection and measurement*

**References**

1. Agiostratidou G, Anhalt H, Ball D, et al. Standardizing clinically meaningful outcome measures beyond HbA1c for type 1 diabetes: A consensus report of the American Association of Clinical Endocrinologists, the American Association of Diabetes Educators, the American Diabetes Association, the Endocrine Society, JDRF International, The Leona M. and Harry B. Helmsley Charitable Trust, the Pediatric Endocrine Society, and the T1D Exchange. Diabetes Care. 2017;40(12): 1622-1630.
2. Allin BSR, Bradnock T, Kenny S, et al. NETS1HD study: Development of a Hirschsprung's disease core outcome set. Arch Dis Child. 2017;102(12):1143-1151.
3. Allin BS, Irvine A, Patni N, et al. Variability of outcome reporting in Hirschsprung’s disease and gastroschisis: a systematic review. Sci Rep. 2016;6:38969.
4. Ammendolia C, Schneider M, Williams K, et al. The physical and psychological impact of neurogenic claudication: The patients' perspectives. J Can Chiropr Assoc. 2017;61(1):18-31.
5. Askenazi DJ, Morgan C, Goldstein SL, et al. Strategies to improve the understanding of long-term renal consequences after neonatal acute kidney injury. Pediatr Res. 2016;79(3):502-508.
6. Avery KNL, Chalmers KA, Brookes ST, et al. Development of a Core Outcome Set for Clinical Effectiveness Trials in Esophageal Cancer Resection Surgery. Ann Surg. 2018;267(4):700-710.
7. Blencowe NS, Strong S, McNair AG, et al. Reporting of short-term clinical outcomes after esophagectomy: a systematic review. Ann Surg. 2012;255:658–666.
8. Barbarot, S., N. K. Rogers, et al. (2016). "Strategies used for measuring long-term control in atopic dermatitis trials: A systematic review." Journal of the American Academy of Dermatology 75(5): 1038-1044.
9. Chalmers, J. R., E. Simpson, et al. (2016). "Report from the fourth international consensus meeting to harmonize core outcome measures for atopic eczema/dermatitis clinical trials (HOME initiative)." British Journal of Dermatology 175(1): 69-79.
10. Gerbens, L. A. A., J. R. Chalmers, et al. (2016). "Reporting of symptoms in randomized controlled trials of atopic eczema treatments: a systematic review." British Journal of Dermatology 175(4): 678-686.
11. Gerbens, L. A. A., C. A. C. Prinsen, et al. (2017). "Evaluation of the measurement properties of symptom measurement instruments for atopic eczema: a systematic review." Allergy 72(1): 146-163.
12. Heinl, D., C. A. C. Prinsen, et al. (2017). "Measurement properties of quality-of-life measurement instruments for infants, children and adolescents with eczema: a systematic review." British Journal of Dermatology 176(4): 878-889.
13. Schmitt, J., S. Langan, et al. (2013). "Assessment of clinical signs of atopic dermatitis: A systematic review and recommendation." Journal of Allergy and Clinical Immunology 132(6): 1337-1347.
14. Spuls, P. I., L. A. A. Gerbens, et al. (2017). "Patient-Oriented Eczema Measure (POEM), a core instrument to measure symptoms in clinical trials: a Harmonising Outcome Measures for Eczema (HOME) statement." British Journal of Dermatology 176(4): 979-984.
15. Benstoem C, Moza A, Meybohm P, et al. A core outcome set for adult cardiac surgery trials: A consensus study. PLoS One. 2017;12(11):e0186772.
16. Benstoem C, Moza A, Autschbach R, et al. Evaluating outcomes used in cardiothoracic
    surgery interventional research: a systematic review of reviews to develop a core outcome set.
    Benedetto U, editor. PLoS One. 2015;10:e0122204.
17. Byrne M, O’Connel A, Egan AM, et al. A core outcomes set for clinical trials of interventions for young adults with type 1 diabetes: an international, multi-perspective Delphi consensus study. Trials. 2017;18: 602.
18. Chiarotto, A., L. J. Maxwell, et al. (2016). "Roland-Morris Disability Questionnaire and Oswestry Disability Index: Which Has Better Measurement Properties for Measuring Physical Functioning in Nonspecific Low Back Pain? Systematic Review and Meta-Analysis." Physical Therapy 96(10): 1620-1637.
19. Christiansen JS, Backeljauw PF, Bidlingmaier M, et al. Growth Hormone Research Society perspective on the development of long-acting growth hormone preparations. Eur J Endocrinol. 2016;174(6):C1-8.
20. de Graaf H, Sukhtankar P, Arch B, et al. Duration of intravenous antibiotic therapy for children with acute osteomyelitis or septic arthritis: A feasibility study. Health Technol Assess. 2017;21(48):1-164.
21. Dohner H, Estey E, Grimwade D, et al. Diagnosis and management of AML in adults: 2017 ELN recommendations from an international expert panel. Blood. 2017;129(4):424-447.
22. D¨ohner H, Estey EH, Amadori S, et al. European LeukemiaNet. Diagnosis and management of acute myeloid leukemia in adults: recommendations from an international expert panel, on behalf of the European LeukemiaNet. Blood. 2010;115(3):453-474.
23. Egan AM, Galjaard S, Maresh MJA, et al. A core outcome set for studies evaluating the effectiveness of prepregnancy care for women with pregestational diabetes. Diabetologia. 2017;60(7):1190-1196.
24. Flint AE, Waterman M, Bowmer G, et al. Neuropsychological outcomes following paediatric temporal lobe surgery for epilepsies: Evidence from a systematic review. Seizure. 2017;52:89-116.
25. Gan, E. Y., V. Eleftheriadou, et al. (2017). "Repigmentation in vitiligo: position paper of the Vitiligo Global Issues Consensus Conference." Pigment Cell & Melanoma Research 30(1): 28-40.
26. Garcia-Cardenas V, Armour C, Benrimoj SI, et al. Pharmacists' interventions on clinical asthma outcomes: a systematic review. Eur Respir J. 2016;47(4):1134-1143.
27. Glynne-Jones, R., R. Adams, et al. (2017). "Clinical endpoints in trials of chemoradiation for patients with anal cancer." Lancet Oncology 18(4): e218-e227.
28. Grieve S, Perez RSGM, Birklein F, et al. Recommendations for a first Core Outcome Measurement set for complex regional PAin syndrome Clinical sTudies (COMPACT). Pain. 2017;158(6):1083-1090.
29. Harris PNA, McNamara JF Lye DC, et al. Proposed primary endpoints for use in clinical trials that compare treatment options for bloodstream infection in adults: a consensus definition. Clin Microbiol Infect. 2017;23(8):533-541.
30. Hernandez Yenty QM, Jurgens WJ, van Zuijlen PP, et al. Treatment of the benign inverted nipple: A systematic review and recommendations for future therapy. Breast. 2016;29:82-89.
31. Højgaard, P., L. Klokker, et al. (2017). "A systematic review of measurement properties of patient reported outcome measures in psoriatic arthritis: A GRAPPA-OMERACT initiative." Seminars in Arthritis and Rheumatism.
32. Ogdie, A., M. De Wit, et al. (2017). "Defining outcome measures for psoriatic arthritis: A report from the GRAPPA-OMERACT working group." Journal of Rheumatology 44(5): 697-700.
33. Kalyoncu U, Ogdie A, Campbell W, Bingham CO 3rd, de Wit M, Gladman DD, et al. Systematic literature review of domains assessed in psoriatic arthritis to inform the update of the psoriatic arthritis core domain set. RMD Open. 2016; 2:e000217.
34. Orbai, A. M., M. De Wit, et al. (2017). "Updating the psoriatic arthritis (PsA) core domain set: A report from the PsA workshop at OMERACT 2016." Journal of Rheumatology 44(10): 1522-1528.
35. Kamat AM, Sylvester RJ, Böhle A, et al. Definitions, End Points, and Clinical Trial Designs for Non-Muscle-Invasive Bladder Cancer: Recommendations From the International Bladder Cancer Group. J Clin Oncol. 2016;34(16):1935-1944.
36. Karam A, Ledermann JA, Kim JW, et al. Fifth Ovarian Cancer Consensus Conference of the Gynecologic Cancer InterGroup: first-line interventions. Ann Oncol. 2017;28(4):711-717.
37. Kenny KP, Day PF, Shairf MO, et al. What are the important outcomes in traumatic dental injuries? An international approach to the development of a core outcome set. Dent Traumatol. 2018;34(1):4-11.
38. Sharif MO, Tejani-Sharif A, Kenny K, et al. A systematic review of outcome measures used in clinical trials of treatment interventions following traumatic dental injuries. Dent Traumatol. 2015;31:422–8.
39. Kilari D, Soto-Perez-de-Celis E, Mohile SG, et al. Designing exercise clinical trials for older adults with cancer: Recommendations from 2015 Cancer and Aging Research Group NCI U13 Meeting. J Geriatr Oncol. 2016;7(4):293-304.
40. Klokkerud M, Dagfinrud H, Uhlig T, et al. Developing and testing a consensus-based core set of outcome measures for rehabilitation in musculoskeletal diseases. Scand J Rheumatol. 2018;47(3):225-234.
41. Kwakkel G, Lannin NA, Borschmann K, et al. Standardized measurement of sensorimotor recovery in stroke trials: Consensus-based core recommendations from the Stroke Recovery and Rehabilitation Roundtable. Int J Stroke. 2017;12(5):451-461.
42. Layton AM, Eady EA, Thiboutot DM, et al. Identifying What to Measure in Acne Clinical Trials: First Steps towards Development of a Core Outcome Set. J Invest Dermatol. 2017;137(8):1784-1786.
43. Mackie, S. L., H. Twohig, et al. (2017). "The OMERACT core domain set for outcome measures for clinical trials in polymyalgia rheumatica." Journal of Rheumatology 44(10): 1515-1521
44. MacLennan S, Williamson PR, Bekema H, et al. A core outcome set for localised prostate cancer effectiveness trials. BJU Int. 2017;120(5B):E64-E79.
45. Marrie RA, Miller A, Sormani MP, et al. Recommendations for observational studies of comorbidity in multiple sclerosis. Neurology. 2016;86(15):1446-1453.
46. McNamara RL, Spatz ES, Kelley TA, et al. Standardized Outcome Measurement for Patients With Coronary Artery Disease: Consensus From the International Consortium for Health Outcomes Measurement (ICHOM). J AM Heart Assoc. 2015;4(5). pii: e001767.
47. Millar AN, Daffu-O'Reilly A, Hughes CM, et al. Development of a core outcome set for effectiveness trials aimed at optimising prescribing in older adults in care homes. Trials. 2017;18(1):175.
48. Murray IR, Geeslin AG, Goudie EB, et al. Minimum Information for Studies Evaluating Biologics in Orthopaedics (MIBO): Platelet-Rich Plasma and Mesenchymal Stem Cells. J Bone Joint Surg Am. 2017;99(10):809-819.
49. Nabbout R, Auvin S, Chiron C, et al. Development and content validation of a preliminary core set of patient- and caregiver-relevant outcomes for inclusion in a potential composite endpoint for Dravet Syndrome. Epilepsy Behav. 2018;78:232-242.
50. Nikiphorou E, Mackie SL, Kirwan J, et al. Achieving consensus on minimum data items (including core outcome domains) for a longitudinal observational cohort study in rheumatoid arthritis. Rheumatology. 2017;56(4):550-555.
51. Obbarius A, van Maasakkers L, Baer L, et al. Standardization of health outcomes assessment for depression and anxiety: recommendations from the ICHOM Depression and Anxiety Working Group. Qual Life Res. 2017;26(12):3211-3225.
52. Ong WL, Schouwenburg MG, van Bommel ACM, et al. A Standard Set of Value-Based Patient-Centered Outcomes for Breast Cancer: The International Consortium for Health Outcomes Measurement (ICHOM) Initiative. JAMA Oncol. 2017;3(5):677-685.
53. Page MJ, Huang H, Verhagen AP, et al. Identifying a core set of outcome domains to measure in clinical trials for shoulder disorders: A modified Delphi study. RMD Open. 2016;2(2):e000380.
54. Buchbinder R, Page MJ, Huang H, et al. A preliminary core domain set for clinical trials of shoulder disorders: A report from the OMERACT 2016 shoulder core outcome set special interest group. J Rheumatol. 2017;44(12):1880-1883.
55. Page MJ, Huang H, Gagnier JJ, et al. Outcome reporting in randomised trials for shoulder conditions:
    literature review to inform the development of a core outcome set.Arthritis Care Res. 2018;70(2):252-259.
56. Rief W, Burton C, Frostholm L, et al. Core outcome domains for clinical trials on somatic symptom disorder, bodily distress disorder, and functional somatic syndromes: European Network on Somatic Symptom Disorders recommendations. Psychosom Med. 2017;79(9):1008-1015.
57. Robson, J. C., G. Tomasson, et al. (2017). "OMERACT endorsement of patient-reported outcome instruments in antineutrophil cytoplasmic antibody-associated vasculitis." Journal of Rheumatology 44(10): 1529-1535.
58. Ruiz CE, Hahn RT, Berrebi A, et al. Clinical trial principles and endpoint definitions for paravalvular leaks in surgical prosthesis: An expert statement. J Am Coll Cardiol. 2017;69(16):2067-2087.
59. Sanderson, T., J. Kirwan, et al. (2016). "Item Development and Face Validity of the Rheumatoid Arthritis Patient Priorities in Pharmacological Interventions Outcome Measures." The Patient: Patient-Centered Outcomes Research 9(2): 103-115.
60. Sharrock AE, Barker T, Yuen HM, et al. Management and closure of the open abdomen after damage control laparotomy for trauma. A systematic review and meta-analysis. Injury. 2016;47(2):296-306.
61. Sreih AG, Alibaz-Oner F, Kermani TA, et al. Development of a core set of outcome measures for large-vessel vasculitis: Report from OMERACT 2016. J Rheumatol. 2017;44(12):1933-1937.
62. Steutel NF, Benninga MA, Langendam MW, et al. Developing a core outcome set for infant colic for primary, secondary and tertiary care settings: A prospective study. BMJ Open. 2017;7(5):e015418.
63. Steutel NF, Benninga MA, Langendam MW, et al. Reporting outcome measures in trials of infant colic. J Pediatr Gastroenterol Nutr. 2014;59:341-346.
64. Stoffel JT, Peterson AC, Sandhu JS, et al. AUA white paper on nonneurogenic chronic urinary retention: Consensus definition, treatment algorithm, and outcome end points. J Urol. 2017;198(1):153-160.
65. Stoner MC, Calligaro KD, Chaer RA, et al. Reporting standards of the Society for Vascular Surgery for endovascular treatment of chronic lower extremity peripheral artery disease: Executive summary. J Vasc Surg. 2016;64(1):227-228.
66. Turnbull AE, Sepulveda KA, Dinglas VD, et al. Core domains for clinical research in acute respiratory failure survivors: An international modified delphi consensus study. Crit Care Med. 2017;45(6):1001-1010.
67. Needham DM, Sepulveda KA, Dinglas VD, et al. Core outcome measures for clinical research in acute respiratory failure survivors. An international modified delphi consensus study. Am J Respir Crit Care Med. 2017;196(9):1122-1130.
68. Dinglas VD, Chessare CM, Davis WE, et al. Perspectives of survivors, families and researchers on key outcomes for research in acute respiratory failure. Thorax. 2018;73(1):7-12.
69. Hashem MD, Nallagangula A, Nalamalapu S, et al. Patient outcomes after critical illness: a systematic review of qualitative studies following hospital discharge. Crit Care. 2016;20(1):345.
70. Turnbull AE, Rabiee A, Davis WE, et al. Outcome measurement in ICU survivorship research from 1970-2013: a scoping review of 425 publications. Crit Care Med. 2016;44(7):1267-1277.
71. Hodgson CL, Turnbull AE, Iwashyna TJ, et al. Core domains in evaluating patient outcomes after acute respiratory failure: international multidisciplinary clinician consultation. Phys Ther. 2017;97(2):167-174.
72. Eakin MN, Patel Y, Mendez-Tellez P, et al. Patient outcomes after acute respiratory failure: A qualitative study of survivors’ experience using the PROMIS framework. Am J Crit Care. 2017;26(6):456-465.
73. Tong A, Gill J, Budde K, et al. Toward establishing core outcome domains for trials in kidney transplantation: Report of the standardized outcomes in nephrology-kidney transplantation consensus workshops. Transplantation. 2017;101(8):1887-1896.
74. Sautenet B, Tong A, Manera KE, et al. Developing Consensus-Based Priority Outcome Domains for Trials in Kidney Transplantation: A Multinational Delphi Survey With Patients, Caregivers, and Health Professionals. Transplantation. 2017;101(8):1875-1886.
75. Howell M, Tong A, Wong G, et al. Important outcomes for kidney transplant recipients: a nominal group and qualitative study. Am J Kidney Dis. 2012;60(2):186-196.
76. Howell M, Wong G, Turner RM, et al. The consistency and reporting of quality-of-life outcomes in trials of immunosuppressive agents in kidney transplantation: a systematic review and meta-analysis. Am J Kidney Dis. 2016;67:762-774.
77. van der Poel HG, Wit EM, Acar C, et al. Sentinel node biopsy for prostate cancer: report from a consensus panel meeting. BJU Int. 2017;120(2):204-211.
78. Acar C, Kleinjan GH, van den Berg NS, et al. Advances in sentinel node dissection in prostate cancer from a technical perspective. Int J Urol. 2015;22:898-909.
79. Walker UA, Clements PJ, Allanore Y, et al. Muscle involvement in systemic sclerosis: points to consider in clinical trials. Rheumatology. 2017;56(suppl_5):v38-v44.
80. Wallace SJ, Worrall L, Rose T, et al. Using the International Classification of Functioning, Disability, and Health to identify outcome domains for a core outcome set for aphasia: a comparison of stakeholder perspectives. Disabil Rehabil. 2017;12:1-10.
81. Wallace SJ, Worrall L, Rose T, et al. Which outcomes are most important to people with aphasia and their families? An international nominal group technique study framed within the ICF. Disabil Rehabil. 2017;39(14):1364-1379.
82. Wallace SJ, Worrall L, Rose T, et al. Which treatment outcomes are most important to aphasia clinicians and managers? An international e-Delphi consensus study. Aphasiology. 2017;31(6):643-673.
83. Wallace SJ, Worrall L, Rose T, et al. Core outcomes in aphasia treatment research: An e-Delphi consensus study of international aphasia researchers. Am J Speech Lang Pathol. 2016;25(4):S729-S742.
84. Warners MJ, Hindryckx P, Levesque BG, et al. Systematic review: Disease activity indices in eosinophilic esophagitis. Am J Gastroenterol. 2017;112(11):1658-1669.
85. Webster L, Groskreutz D, Grinbergs-Saull A, et al. Development of a core outcome set for disease modification trials in mild to moderate dementia: A systematic review, patient and public consultation and consensus recommendations. Health Technol Assess. 2017;21(26):1-192.
86. Webster L, Groskreutz D, Grinbergs-Saull A, et al. Core outcome measures for interventions to prevent or slow the progress of dementia for people living with mild to moderate dementia: Systematic review and consensus recommendations. PLoS One. 2017;12(6):e0179521.
87. Williams MR, Ward DS, Carlson D, et al. Evaluating Patient-Centered Outcomes in Clinical Trials of Procedural Sedation, Part 1 Efficacy: Sedation Consortium on Endpoints and Procedures for Treatment, Education, and Research Recommendations. Anesth Analg. 2017;124(03):821-830.
88. Wolters, P. L., S. Martin, et al. (2016). "Patient-reported outcomes of pain and physical functioning in neurofibromatosis clinical trials." Neurology 87(7 Suppl 1): S4-S12.
